# Spatial Distribution Analysis and Comparative Forecasting of Dengue Resurgence in the Philippines (2025–2027): A Nationwide Study

**DOI:** 10.1155/tbed/7480710

**Published:** 2025-10-15

**Authors:** Kenny Oriel Aranas Olana, Napaphat Poprom, Pallop Siewchaisakul, Veerasak Punyapornwithaya, Aksara Thongprachum

**Affiliations:** ^1^Doctor of Public Health Program, Faculty of Public Health, Chiang Mai University, Chiang Mai, Thailand; ^2^Department of Veterinary Paraclinical Sciences, Faculty of Veterinary Medicine, Visayas State University, Baybay, Leyte, Philippines; ^3^Faculty of Public Health, Chiang Mai University, Chiang Mai, Thailand; ^4^Research Center for Veterinary Biosciences and Veterinary Public Health, Faculty of Veterinary Medicine, Chiang Mai University, Chiang Mai, Thailand; ^5^Veterinary Public Health and Food Safety Centre for Asia Pacific (VPHCAP), Faculty of Veterinary Medicine, Chiang Mai University, Chiang Mai, Thailand

**Keywords:** Dengue, forecasting, NNAR, Philippines, time series

## Abstract

Prediction of dengue continues to be valuable in endemic countries. Time series forecasting methods have been widely employed for predicting future dengue trends and outbreaks. The study aimed to determine the spatial distribution, trends, and seasonality of dengue cases and compare the predictive accuracy of seasonal autoregressive integrated moving average (SARIMA), neural network autoregression (NNAR), random forest (RF), long–short term memory (LSTM), trigonometric exponential smoothing state–space model with Box–Cox transformation, ARMA errors, trend and seasonal components (TBATS), and Prophet in forecasting dengue cases in the Philippines. Monthly data from 2017 to 2024 across all provinces were obtained and were partitioned into training (January 2017–December 2023) and testing segments (January 2024–December 2024). Model performance was assessed by analyzing the training data using time series techniques and comparing the resulting forecasts with empirical values from the test dataset. In total, 3-year projections were generated by implementing the models on the entire dataset. The study analyzed 1,903,425 dengue cases with a mean monthly incidence of 17.66 ± 15.97 per 100,000 population. Regular seasonal epidemics were identified, peaking from July to September. NNAR outperformed the other models and predicted an annual average of 444,678 cases from 2025 to 2027. This is the first study to apply SARIMA, RF, LSTM, TBATS, and Prophet in forecasting dengue cases in the Philippines at a national scale. The study offers new insights into disease forecasting, particularly in the application of advanced time series methodologies. These findings should be considered to strengthen surveillance, prevention, and control against dengue.

## 1. Introduction

Dengue is a mosquito-borne, viral emerging infectious disease caused by dengue virus (DENV) of the Family Flaviviridae [1]. It is an epidemic-prone disease that risks more than 3.9 billion people in over 129 countries worldwide [2]. The clinical presentation of dengue spans from mild fever to severe manifestations, such as dengue hemorrhagic fever (DHF) and dengue shock syndrome (DSS), with key diagnostic features including thrombocytopenia, leucopenia, and increased permeability of blood vessels. Common symptoms of dengue include fever, headaches, pain behind the eyes, muscle and joint pain, nausea/vomiting, rash, and fatigue [1, 3]. While clinical manifestations resembling dengue were first described in Madras, India in 1780, virological confirmation of a dengue fever (DF) epidemic was not achieved until 1963–1964, when an outbreak occurred in Calcutta and India's eastern coastal regions. The initial recognition of DHF as a distinct clinical entity occurred in the Philippines in 1953, and further DHF cases were then detected in other Southeast Asian countries and the Western Pacific region [4]. As part of the United Nations Sustainable Development Goals (SDGs), a global initiative aims to halt epidemics of neglected tropical diseases, specifically including dengue and malaria, before 2030; however, the world is far from meeting the milestones set in 2020. The incidence of mosquito-borne diseases has been continually increasing globally due to the geographic expansion of vectors across continents consequent to climate change, heat waves, and flooding, aided by global trade, travel, and disease introduction to new areas [5, 6].

Still, the Philippines experiences an exceptionally high dengue disease burden, ranking among the most severely affected countries in the region. In 2023 alone, the country recorded a total of 195,603 dengue cases, which is significantly higher than its neighboring countries such as Viet Nam (166,619), Thailand (150,808), Malaysia (120,418), Indonesia (114,720), Lao PDR (31,997), and Cambodia (31,567) [7]. The Southeast Asian region constitutes the majority (75%) of the total burden of DF globally, and the Philippines contributes greatly to this burden [3, 8]. The heterogeneity in dengue case definitions and reporting systems across countries, especially those focusing on severe or hospitalized cases, makes direct numerical comparisons challenging. Nevertheless, the disease remains one of the country's most significant public health concerns, demanding urgent attention [9, 10]. With the current standing population in the Philippines, the susceptible fraction is not clearly defined, and there is no effective available vaccine against DENV serotypes. Control measures are centered on vector control and reducing human–vector contact. These conditions necessitate the forecasting of future case counts and potential fatalities [11, 12, 13]. However, research is scarce in modeling disease burden, describing spatiotemporal patterns and trends, and forecasting of dengue outbreaks in the Philippines. Despite being considered the most well-studied mosquito-borne disease in the country, systematic reviews show only three modeling studies conducted in the Philippines for the past 60 years on dengue research and one for spatial modeling for dengue risk maps [14, 15]. In light of the previous studies in the Philippines [16, 17, 18, 19, 20], there is a need to explore other advanced statistical and machine learning tools to account for complexities in the data and utilize recent data to create an updated forecast as well as to widen the geographic scope to a national scale.

Recent dengue modeling approaches reflect a transition away from traditional statistical methodologies, such as seasonal autoregressive integrated moving average (SARIMA), Poisson regression, linear regression, and generalized additive models, to robust machine learning tools such as neural networks, support vector machine (SVM), random forest (RF), gradient boosting, and long–short term memory (LSTM). In this study, we chose to compare SARIMA, representing the classical statistical technique, with machine learning techniques proven to have superior performances in forecasting dengue incidence, including neural network autoregression (NNAR) [21], RF [22, 23, 24] and LSTM [25, 26, 27]. In addition, we assessed the usefulness of trigonometric exponential smoothing state–space model with Box–Cox transformation, ARMA errors, trend and seasonal components (TBATS) and Prophet time series methodologies as these novel methods were unexplored in the previous studies on dengue forecasting. SARIMA has gained prominence in public health forecasting due to its capacity to process time-dependent data relationships while considering seasonal fluctuations, extended trends, and time lags in time series analyses [20, 28]. However, the technique is insufficient to model nonlinear relationships and is useful only in modeling linear relationships in time series with singular seasonality [29]. Thus, it is insufficient to meet complex forecasting requirements [22]. Since the inception of forecasting using neural networks, only Chakrabotry et al. [21] had utilized NNAR to forecast dengue incidence. As a nonlinear autoregressive (AR) model, NNAR demonstrates several advantages over ANN, SVM, and LSTM approaches, including simplified architecture, clearer interpretation of results, and superior predictive capabilities in numerous applications [28]. RF was used in recent studies to forecast dengue cases in Brazil [24], Columbia [22], and the Philippines [16, 18]. Despite RF's advantageous ability to apply nationally aggregated models to smaller spatial scales in dengue forecasting, a significant limitation is its inability to generate predictions beyond the scope of its training data range [22]. LSTM was found to have the best performance in forecasting dengue cases in studies from India [27], Sri Lanka [26], and China [25]. It can deal with longer dependencies in sequences and incorporate information from extended historical periods in its prediction [30]. Leung et al. [31] systematically reviewed the current practices in predicting dengue outbreaks, all the studies reviewed performed multivariate forecasting despite the discovery of advanced methods such as TBATS and Prophet. A summary of the models used in the present study, including their applications, assumptions, and limitations, is provided in Supporting Information 1: Table S1.

With the advent of advanced statistical and machine learning methodologies, there is a gap in knowledge on the application and effectiveness of these methods in univariate forecasting of dengue cases. To address this gap, we evaluated the predictive efficacy of SARIMA as the baseline method, in comparison with advanced time series models, including NNAR, RF, LSTM, TBATS, and Prophet. Additionally, we utilized national scale data to forecast for the whole country.

The study aimed to determine the spatial distribution, trends, and seasonality of dengue in the Philippines and provide a prospective estimate of dengue occurrences for the next 3 years. The study advances the body of knowledge on forecasting dengue outbreaks and the application of advanced time series methodologies. Furthermore, the study's findings will provide an updated forecast at the national level necessary for the planning, resource allocation, and implementation of disease control and prevention.

## 2. Materials and Methods

### 2.1. Philippines

The Philippines is a country located in Southeast Asia with a population of more than 115 million [32]. It consists of three main islands, Luzon, Visayas, and Mindanao, with Luzon Island being the largest. Overall, the archipelago encompasses approximately 7641 islands [33]. The Philippine climate is influenced by the Northeast Monsoon (December to April) and Southwest Monsoon (May to November) and is classified as tropical marine [34]. The monsoons generally define the country's wet and dry seasons. *Aedes aegypti* is the main vector responsible for dengue transmission in the Philippines, which is predominant in urban areas, *Aedes albopictus* serves as a secondary rural vector [35, 36]. All four serotypes of DENV (1–4) are present in the Philippines [37]. DENV-2 as cosmopolitan genotype in the Philippines, as it continues to circulate in metropolitan areas [38].

### 2.2. Data

The number of reported dengue cases in the Philippine provinces and chartered cities from January 2017 to December 2024 was obtained from the Philippine Department of Health, Epidemiology Bureau. The bureau follows the case classification from the Manual of Procedures for the Philippine Integrated Disease Surveillance and Response 2014 (following the WHO [39] Guidelines 1997): suspect, probable, and confirmed cases [40,]. Suspect case refers to a previously well person with acute febrile illness of 2–7 days duration with clinical signs of dengue. A probable case, on the other hand, refers to a suspect case with a laboratory test, at least a complete blood count (CBC) showing leucopenia with or without thrombocytopenia, and/or Dengue NS1 antigen test, or an immunoglobin antibody test (i.e., IgM ELISA test). A case is confirmed when tested positive in viral culture isolation or polymerase chain reaction (PCR). Cases detected by the Hospital Epidemiology and Surveillance Units (HESU) had to be reported and validated at the provincial or city epidemiology and surveillance units through the Philippine Integrated Disease Surveillance and Response Information System (PIDSR-IS). All reports were then transmitted and underwent a second round of validation at the Regional Epidemiology and Surveillance Units before submission to the national-level Epidemiology Bureau. The Research Institute for Tropical Medicine (RITM), as the national reference laboratory, is looped in the validation flow as a confirmatory laboratory test for some notifiable diseases that can only be done in their facility. Results are then uploaded to the same information system. Estimated human populations by province and by month were derived using the population growth rate from 2015 to 2020 census [41]. Monthly population growth rates were calculated and then applied to population estimation using the exponential population projection formula. Monthly incidence per 100,000 population per province was computed by dividing the monthly reported dengue cases by the estimated population. The entire population of the province was considered as the population at risk.

### 2.3. Descriptive Statistics and Mapping

Frequency distributions, measures of central tendency, and dispersion were computed. The distribution of dengue cases and incidence by month was analyzed using column and line graphs. Cumulative incidence in each province was generated yearly from 2017 to 2024 using the total number of reported dengue cases and the median population of the respective year. This estimate was categorized using quintiles and mapped using the Philippine administrative boundary shapefiles from the National Mapping and Resource Information Authority [42] in QGIS version 3.36 Maidenhead [43] to determine the spatial distribution of the disease in the country.

### 2.4. Time Series Data Preprocessing

Time series data encompassing January 2017 to December 2024 was derived by summing the number of dengue cases by month. This formed two-column data with the date (year–month) as the first column and the number of cases in the second column. These data were then converted to time series objects utilizing *ts*() base R function for subsequent analyses. Lagged values of dengue cases were created for the RF model as features. Prior to LSTM modeling, the data was normalized by way of scaling.

### 2.5. Seasonal and Trend Decomposition Using LOESS (STL) Decomposition, Stationarity, and Seasonality of Time Series Data

To describe the trends and seasonality components of the dengue time series, an STL was carried out using R statistical software version 4.3.3 [44]. STL employs a locally weighted regression approach, specifically LOESS, to estimate the trend and seasonal components within a time series [28]. Through this method, the time–series data were decomposed into three main components: trend, seasonal, and residual, as represented below:

  $$\begin{array}{c} Y_{t}=T_{t}+S_{t}+R_{t} \end{array}$$

where at time *t*, *Y*_*t*_ represents the number of dengue cases, *T*_*t*_ is the trend component of the time series, *S*_*t*_ is the estimated seasonal component, and *R*_*t*_ is the remaining component of the time series. This was done in R using the *stl*() function.

The Augmented Dickey–Fuller (ADF) test was used to assess the stationarity of the time series data with nonstationarity as the null hypothesis [28]. The test is important, as it may entail the necessity to apply for differencing of the series (to achieve stationarity) before model development of time series methods requiring stationarity (e.g., SARIMA).

Ollech and Webel's combined seasonality test (WO-test) was utilized to confirm the data's inherent seasonality. The test combines the QS-test and Kruskal–Wallis (KW) test results, calculated on the residuals of an automatic nonseasonal autoregressive integrated moving average (ARIMA) model [45]. If the *p*-value of the QS-test and KW-test is < 0.01 or <0.002, respectively, the WO-test will classify the corresponding time series as seasonal. This was done using the *combined_test* function of the “seastest” R package. Further details of the test can be found in [45].

### 2.6. Analytical and Modeling Procedure

**Figure 1:**
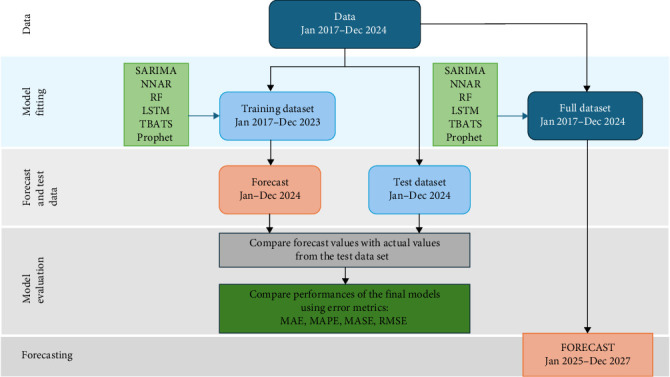
Modeling Procedure. The full dataset of dengue cases was split into training and test datasets. Forecast models—seasonal autoregressive integrated moving average (SARIMA), neural network autoregression (NNAR), random forest (RF), long-short term memory (LSTM), trigonometric exponential smoothing state–space model with Box–transformation, ARMA errors, trend and seasonal components (TBATS), and Prophet model were developed. With the validation data, error measures, including mean absolute error (MAE), mean absolute percent error (MAPE), mean absolute scaled error (MASE), and root mean squared error (RMSE) were determined to compare the performances of prediction models.

Prior to modeling, the time series data was split into a training dataset (January 2017 to December 2023), and a test dataset (January 2024 to December 2024) (Figure 1). The modeling procedure of the study consists of two phases. First, a model from each time series method was finalized using the training dataset, and subsequently evaluated for its performance using the test dataset (out-of-sample data). This step identifies the highest-performing model. By applying the models to the training dataset, forecasts were derived for January 2024 to December 2024, the same period as to the test dataset. These forecast values were then compared with the actual values of the test dataset to assess the model's forecasting performance. Second, the highest-performing model was then utilized to predict future dengue cases based on the full dataset. Furthermore, to ensure an up-to-date forecast, all the time series models were applied to the full dataset to forecast the following 3 years (2025–2027).

All data management, including organization, decomposition, and segmentation was executed through the R statistical software version 4.3.3 [44], leveraging the “tseries” and “forecast” packages. For graphical representation, the packages used were “ggplot2,” “ggfortify,” “plotly,” and “scales”. Details of the functions and packages used in the time-series models are comprehensively described in Hyndman et al. [46].

### 2.7. Time–Series Models

#### 2.7.1. SARIMA Model

SARIMA model is specifically designed to analyze and forecast univariate time series data exhibiting seasonal patterns. The model generates predictions utilizing only the historical values of the target series and inherently includes lagged terms in its components. It extends the traditional ARIMA framework by incorporating three additional hyperparameters that characterize the seasonal component: seasonal autoregression, seasonal differencing, and seasonal moving average. The model also includes a parameter to define the seasonality period. The SARIMA model is conventionally denoted by the following expression [28]:

  $$\begin{array}{c} \phi_{P}\left(B\right)\Phi_{p}\left(B^{s}\right){\left(1-B\right)}^{d}{\left(1-B^{s}\right)}^{D}x_{t}=\theta_{Q}\left(B\right)\Theta_{Q}\left(B^{s}\right)\omega_{t}. \end{array}$$

The model parameters *φ* and *θ* correspond to the nonseasonal AR and moving average components, respectively, while *Φ* and *Θ* denote their seasonal counterparts. The complete SARIMA model is formally expressed as ARIMA (*p*, *d*,*q*)(*P*, *D*,*Q*)_S_, where the nonseasonal components are characterized by *p* (AR order), *d* (differencing degree), and *q* (moving average order). The seasonal elements are defined by *P* (seasonal AR order), *D* (seasonal differencing degree), and *Q* (seasonal moving average order), with *S* representing the seasonal periodicity of the time series.

The development of the SARIMA model was carried out using the *auto.arima*() function from a “forecast” R package. This function utilizes the Hyndman–Khandarkar algorithm for automatic ARIMA modeling through which it determines the *p*, *d*, *q*, *P*, *D*, *Q* for several candidate models, and the optimal model was determined as having yielded the lowest Akaike information criterion (AIC). In addition, autocorrelation function (ACF) and partial ACF were utilized to confirm the *q* and *p* terms, respectively (Supporting Information 2: Figure S1). Residuals of the model were given in Supporting Information 2: Figure S2.

#### 2.7.2. NNAR Model

The NNAR forecasting methodology comprises two sequential phases: initial determination of the AR order, followed by neural network training using the predetermined order and training dataset. The number of input nodes in the neural network structure was determined by the AR order, which corresponds to the time series lags. The notation NNAR(*p*,*k*) designates a model with *p* lagged inputs and *k* hidden layer nodes. For time series exhibiting seasonality, the NNAR model specification takes the form [28] as follows:

  $$\begin{array}{c} \text{NNAR}{\left(p,P,k\right)}_{m}. \end{array}$$

In this specification, *p* represents the number of nonseasonal lagged inputs in the linear AR process, while *P* denotes the seasonal AR lags. The model architecture is further defined by *k*, which specifies the number of neurons in the hidden layer, and *m*, which characterizes the seasonal periodicity of the time series. The number of lagged inputs was specified during modeling. To fit the model, the *nnetar* function in the “forecast” package of R was used. A systematic grid search was conducted to identify the optimal NNAR model configuration, evaluating all parameter combinations based on information criteria (AIC) and prediction accuracy metrics (root mean squared error [RMSE] and mean absolute error [MAE]).

#### 2.7.3. RF Model

RF is an ensemble learning algorithm that leverages multiple decision trees to generate an optimized composite model [47]. The methodology implements bootstrap aggregation (bagging), wherein numerous independent decision trees are trained on random subsets of the training data. The final prediction is derived through the aggregation of individual tree outputs using their mean values. The algorithm quantifies variable importance by evaluating the magnitude of prediction error increment associated with each predictor variable. For univariate forecasting using RF, there is a need to create lagged features. In the study, lagged dengue cases (12 lags) were used as predictors. To construct the RF model, the “*random forest*” and “*dplyr*” R packages were used. To select for the optimal configuration of the RF model, hyperparameters such as the number of trees, mtry, splitrule, and node size were tuned and cross-validated via custom tuning function using the “ranger” package. The configuration that yielded the lowest mean RMSE was chosen.

#### 2.7.4. LSTM Model

LSTM represents a specialized recurrent neural network (RNN) architecture developed by Hochreiter and Schmidhuber [48] in 1997 to overcome the limitations of conventional RNNs, specifically addressing the vanishing and exploding gradient phenomena that compromise model performance. It can learn long-term dependencies, and unlike other RNNs, LSTM adds memory blocks instead of regular neural units (i.e., hidden layers). A common LSTM memory block consists of a cell state and three gates—an input gate, a forget gate, and an output gate, which are expressed in the following notations:

  $$\begin{array}{c} f_{j}=\sigma \left(w_{f}\cdot \left[h_{j-1},x_{j}\right]+b_{f}\right) \end{array}$$

  $$\begin{array}{c} i_{j}=\sigma \left(w_{i}\cdot \left[h_{j-1},x_{j}\right]+b_{i}\right) \end{array}$$

  $$\begin{array}{c} o_{j}=\sigma \left(w_{o}\cdot \left[h_{j-1},x_{j}\right]+b_{o}\right) \end{array}$$

  $$\begin{array}{c} \hat{C}=\tanh\left(w_{c}\cdot \left[h_{j-1},x_{j}\right]+b_{c}\right) \end{array}$$

  $$\begin{array}{c} C_{j}=f_{j}\;*\;C_{j-1}+i_{j}\;*\;\hat{C} \end{array}$$

  $$\begin{array}{c} h_{j}=o_{j}\;*\;\tanh\left(C_{j}\right). \end{array}$$

The LSTM architecture comprises three regulatory gates: the forget gate (*f*), input gate (*i*), and output gate (*o*). The input gate regulates information flow into the cell state, the forget gate modulates the retention and elimination of cellular information, and the output gate is used to decide output from the cell. 'σ' represents the logistic sigmoid function. *w*, *b*, and *h* are weights, biases, and values of hidden layers, respectively. $\hat{C}$ and C are vectors of new candidate values and cell state, respectively. To develop the model, “keras3” and “tensorflow” R packages were used. A comprehensive grid search methodology was employed to optimize model hyperparameters, with model selection based on cross-validated mean squared error (MSE) performance. The hyperparameter optimization space included neural network architecture parameters (LSTM hidden units), regularization parameters (dropout rate), training parameters (learning rate, batch size), and convergence parameters (training epochs).

#### 2.7.5. TBATS Model

TBATS is a generalization of the traditional seasonal models with multiple seasonal periods developed by De Livera, Hyndman and Snyder in 2011 [49]. This model represents an enhanced version of BATS (Box–Cox transform, ARMA errors, Trend, and Seasonal components), with modifications to handle multiple seasonality patterns. The model incorporates a trigonometric framework that effectively captures seasonal patterns, accounts for residual autocorrelation, and accommodates the nonlinear characteristics inherent in empirical time series data. The model is represented as [28]:

  $$\begin{array}{c} \text{TBATS}\left(\omega ,p,q,\varphi ,\left\{m_{1},k_{1}\right.,\left\{m_{2},k_{2}\right\},\ldots ,\left\{m_{T},k_{T}\right\}\right), \end{array}$$

where *p* and *q* characterize the ARMA process, while *m*_1_ through *m*_*T*_ define the multiple seasonal periodicities. The seasonal component is further parameterized by *k*, which denotes the number of allocated harmonics. The model specification is completed by *ω*, which parameterizes the Box–Cox transformation, and *φ*, which quantifies the dampening effect. To enhance model parsimony, the seasonal components are represented through trigonometric functions derived from Fourier series. The model was done using the *tbats*() function included in the “forecast” R package. This function automatically optimizes several components of the model to best fit the data by selecting the configuration with the lowest AIC. The optimization includes determining whether to apply Box–Cox transformation, ARMA error correction, trend damping, and the optimal values for all smoothing parameters.

#### 2.7.6. Prophet Model

Prophet is a nonlinear regression model utilizing a decomposable time series structure that integrates trend, seasonal, and holiday components. The model incorporates dual seasonal patterns: a yearly seasonal component modeled using the Fourier series (which creates a more flexible model for periodic effects) and a weekly seasonal component modeled using dummy variables. This is expressed as [28]:

  $$\begin{array}{c} y_{t}=g\left(t\right)+s\left(t\right)+h\left(t\right)+\varepsilon_{t}, \end{array}$$

where *g*(*t*) describes a piecewise-linear trend (or “growth term”), the knots (or changepoints) for the piecewise-linear trend are automatically selected if not explicitly specified; *s*(*t*) describes the various seasonal patterns, the seasonal component consists of Fourier terms of the relevant periods. By default, order 10 is used for annual seasonality. *h*(*t*) captures the holiday effects, holiday effects are added as simple dummy variables, and *ε*_*t*_ is a white noise error term. The model is estimated using a Bayesian approach to allow for automatic selection of the changepoints and other model characteristics. The model was constructed using the *prophet*() function of the “prophet” R package.

### 2.8. Model Performance Evaluation and Forecasting

Time-series models developed from the training data (2017–2023) were used to predict dengue cases in 2024. To evaluate forecasting performance, we compared the models' predictions with the actual out-of-sample values and calculated corresponding error metrics. Multiple performance metrics were utilized to compare the predictive accuracy of models constructed using the training data. These metrics include MAE, mean absolute percent error (MAPE), mean absolute scaled error (MASE), and RMSE, which are fully discussed by Hyndman and Anthanasopoulos [28]. The lower error metrics values indicate better model performance. To ensure optimal model configuration for each technique, the models were selected based on automatic selection for SARIMA, TBATS, and Prophet, and based on the results of a grid search for NNAR, RF, and LSTM.

To leverage all available data, the refined models were implemented on the comprehensive dataset. This strategy permitted the generation of 3-year dengue case forecasts extending from the study's final recorded observation.

## 3. Results

### 3.1. Descriptive Analysis

The data spans 96 months, from January 2017 to December 2024, with a total of 1,903,425 dengue cases recorded in the entire period. The annual number of dengue cases in the Philippines ranged from 80,919 to 437,089, with a median of 233,136 (Interquartile range [IQR] = 136,278, 301,782). Mean monthly dengue incidence averaged 17.66 ± 15.97 (mean ± standard deviation) per 100,000 population. For a given month in a specific province, a median estimate of 65 (IQR, 21, 181) dengue cases can be observed.

### 3.2. Spatial Distribution

**Figure 2:**
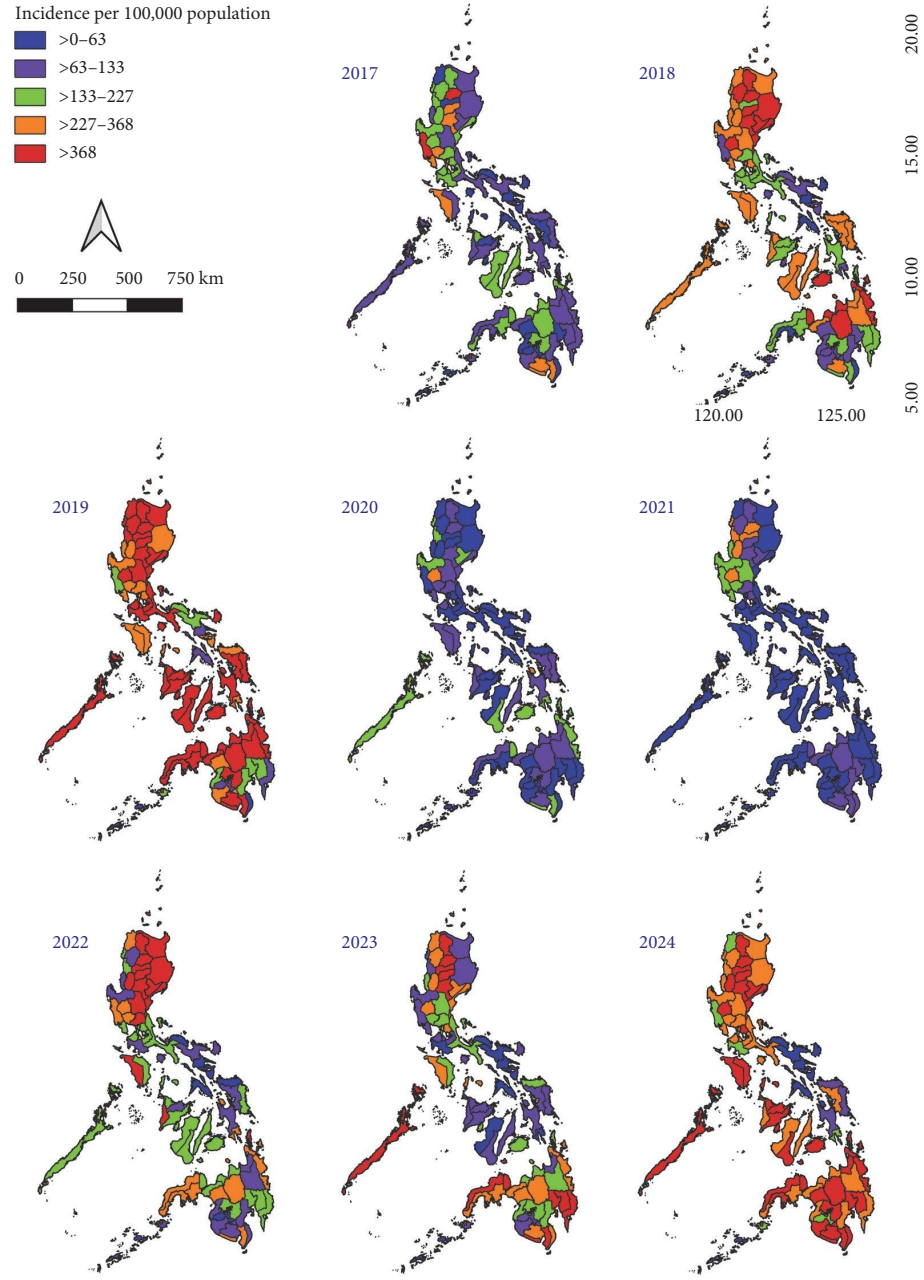
Spatial distribution of dengue incidence by province in the Philippines from 2017–2024.

Dengue occurred in all the administrative provinces of the archipelago.  Figure 2 shows the spatial distribution of dengue incidence by province from 2017 to 2024. Cumulative incidence was computed across the years of the study period using the annual number of dengue cases and the median population of each province. All the provinces and chartered cities were affected by dengue over the years, with some variations in incidence. The ranking of provinces and major cities with the highest incidences was given in Supporting Information 1: Table S2. Notably, an epidemic occurred in 2019 [50] that heavily impacted the country, with 70.25% (85/121) of the epidemiology surveillance units reporting cases exceeding the 300 cases per 100,000 population mark. During this event, the highest incidences were observed in the following areas: Guimaras (1350), Iloilo (1178), Tacloban City (1136), Apayao (1118), Zamboanga Sibugay (994), and Aklan (981). Interestingly, a great resurgence of similar magnitude has occurred in 2024, with the highest incidence recorded in the following areas: Baguio City (2328), Mountain Province (2258), Apayao (1560), Benguet (1264), and Quezon City with 1250 cases per 100,000 population. Only Puerto Princesa City (368) and the province of Siquijor (374) had surpassed 300 cases per 100,000 population in 2020, and Baguio City (369) and Kalinga (309) in 2021.

### 3.3. Temporal Distribution, Trends, and Seasonality

**Figure 3:**
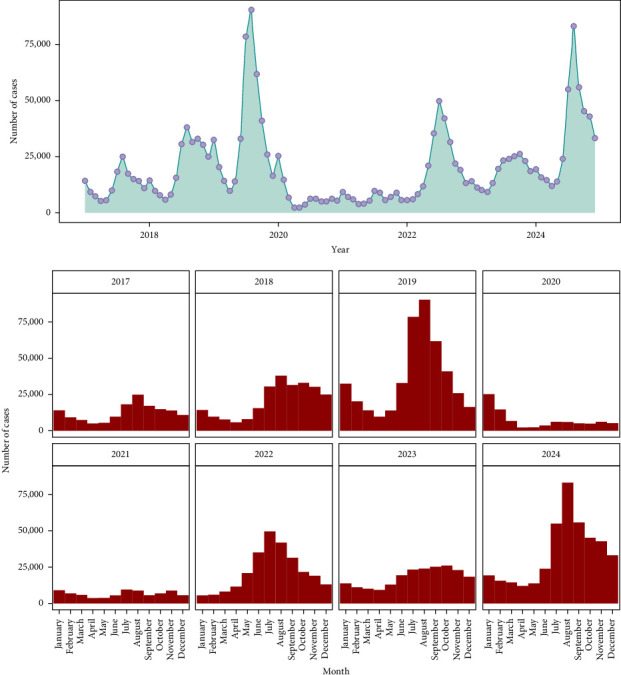
Distribution of dengue cases in the Philippines by month–year, 2017–2024.

**Figure 4:**
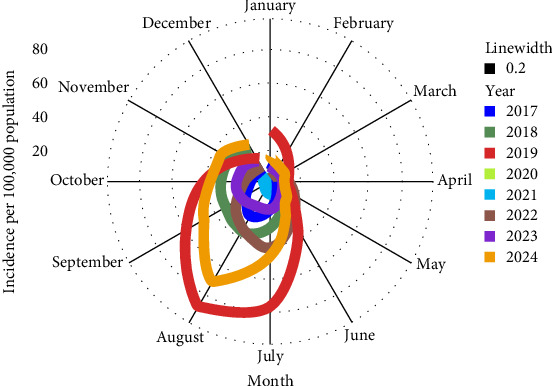
Seasonal plot of dengue incidence by month–year from 2017–2024.

Annual and monthly temporal distributions of dengue cases are shown in Figure 3. The highest record of dengue cases was in 2019, reaching 437,089, followed by 2024 with 413, 960, while the lowest record was in 2021, with 80,919 cases. Anomalies identified with a very high number of cases were data points: July (78,436), August (90,284), and September (61,711) of 2019; and August of 2024 with 83,135 cases (Supporting Information 2: Figure S3). The disease occurs throughout the year, but seasonality is notable, with monthly dengue incidence that often peaks between July and September (Figure 4).

**Figure 5:**
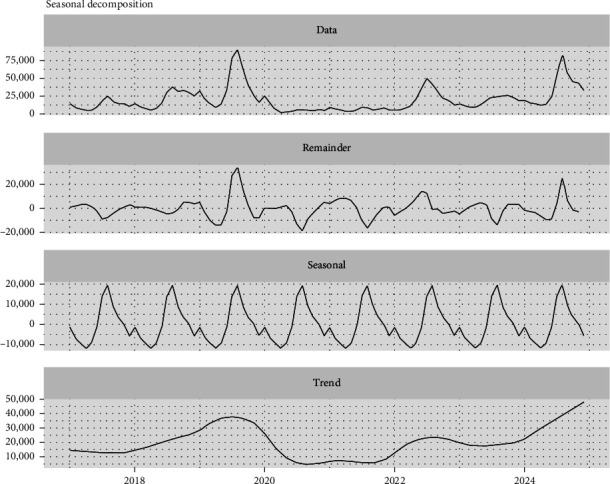
Decomposition of data.

The trend, seasonal patterns, and residual components of dengue cases are shown in Figure 5. Analysis of temporal trends shows a trajectory beginning in December 2017 to August 2019, followed by a decline from October 2019 to May 2020. It then stabilizes until September 2021 and trends upward from October 2021 to September 2022. It slightly trended down to 2023, stabilized and then trended upward to 2024. The ADF test confirms the data is nonstationary (*p*-value = 0.2949) with a lag order equal to four to account for autocorrelation. The strong seasonality of the data was further confirmed by the WO test, which gave a combined test *p*-value equal to < 0.0001, and as shown by the seasonal component of the decomposition. The remainder represents the random component of the time series data and displays increased variability in the years 2019 and 2024.

### 3.4. Time–Series Models, Performances, and Forecasts

The SARIMA model, ARIMA (3,0,0)(1,0,1)_12_, was identified by the software as the best-fitting model that yielded the lowest AIC with no violations (Ljung-Box test *p*-value = 0.5934). Residuals of the model are normally distributed, and the ACF indicates no significant departure from white noise with zero mean (Supporting Information 2: Figure S2). The model has three nonseasonal AR orders, and the seasonal component has seasonal AR and moving average order terms with a period of 12. The model indicated no trend and seasonal differencing needed. The best forecasting model for NNAR was NNAR(4,1,5)_12_, which is defined as a NNAR model using four lagged inputs, with one hidden layer containing five neurons, accounting for seasonality with a period of 12. The model captures both the short-term dependencies (through the four lagged inputs) and the seasonal patterns. The optimal RF model configuration identified consists of 1000 trees, an mtry value of 8, and a node size of 1. The most important features were lag 1 and lag 11. For the LSTM model, the optimal model comprised of a layer with 32 units, four timesteps, a batch size of 8, learning rate equal to 0.01, and 100 epochs. The best-fitting TBATS model is TBATS(0.0082, [,], [ < 12,5>]), suggesting the data is almost log-transformed, has no AR term and two moving average terms, no damped trend, a seasonal component of 12 periods suggesting annual seasonality, and five Fourier terms or harmonics used to model the seasonality. For the Prophet model, the best fit was achieved with a linear growth trend, automatic selection of yearly seasonality, a seasonality prior scale of 10, a changepoint prior scale of 0.05, a holiday prior scale of 10 and 25 changepoints.

**Figure 6:**
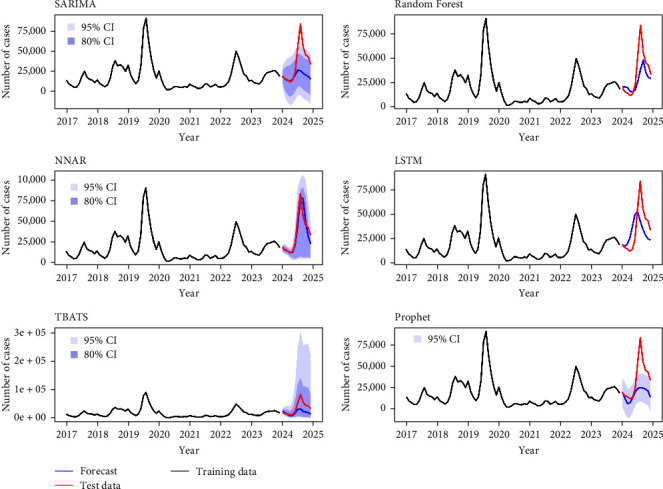
Model performance. Depiction of actual values from the test dataset and forecast values from the training dataset. CI, represents the confidence interval of the forecast.

The machine learning models graphically show promising performances in predicting the test data.  Figure 6 depicts the actual values encompassing the training (January 2017–December 2023) and the test datasets (January 2024 to December 2024) incorporated with the forecast values (January 2024 to December 2024) of the final models derived from the training data. NNAR forecasts align more accurately with the test data, while the LSTM and RF models also generally follow the test data trend, albeit with less precision. The confidence intervals of NNAR depict only positive values in its forecast, while SARIMA and the Prophet intervals go below zero.

Error metrics applied to evaluate the models are presented in Table 1. All models exhibited degraded performance when evaluated on unseen test data, with error metrics consistently higher than those observed during training. Given that test set error metrics represent real-world predictive performance, these measures constitute the appropriate basis for model selection. The evaluation of six forecasting models on the test dataset revealed significant variation in predictive performance across all error metrics. Overall, NNAR outperformed all other models conducted in this study, achieving the lowest test errors across all measures. Both NNAR and RF achieved MASE values below 1.0, indicating superior performance compared to a seasonal naive benchmark, while the other models performed worse than this simple baseline (MASE > 1.0). This indicates that NNAR and RF showed acceptable generalization capability. These results suggest that for this particular dataset, machine learning approaches such as NNAR and RF outperformed both complex deep learning approaches and sophisticated statistical time series methods when applied to previously unseen data.

**Figure 7:**
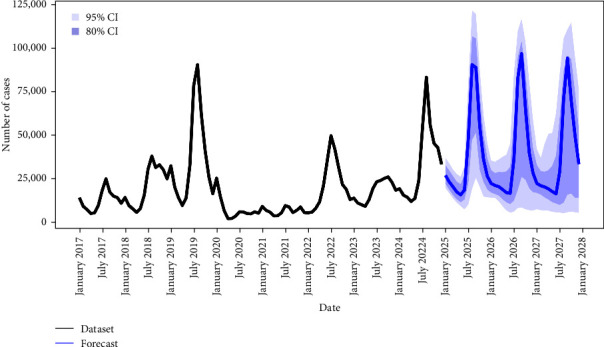
Forecast of monthly dengue cases in the Philippines from 2025 to 2027. CI, represents the confidence interval of the forecast.

A substantial number of dengue cases is forecasted in the coming years.  Figure 7 presents the forecasted number of dengue cases for 2025–2027, as predicted by the NNAR model applied to the full dataset. Based on the mean forecast of the six models, the total number of cases forecasted until 2027 is 1,289,331. For 2025, 2026, and 2027, the predicted annual numbers of cases were 423,455, 435,416, and 430,459, respectively. Further details are presented in Supporting Information 1: Table S3 and Supporting Information 2: Figure S4.

The NNAR model, having the superior performance, predicted a higher number of cases than the mean forecast of all six models. It estimated an annual average of 444,678 cases from 2025 to 2027, which renders a monthly average of 37,057 cases, ranging from 17,058 to 95,466 cases.

## 4. Discussion

Dengue is an acute viral disease endemic in the Philippines that impacts all regions and populations. The ambiguity of the magnitude of the susceptible population in the country necessitates forecasting of the number of prospective cases or fatalities. Results of the study indicate dengue transmission has been documented across all administrative provinces of the archipelago, underscoring its widespread geographic distribution. The implemented machine learning models demonstrated robust predictive performance when evaluated against test data, and their forecasts indicate a substantial increase in dengue cases in the forthcoming years. These findings highlight the importance of utilizing advanced time-series methodologies to forecast dengue cases in the coming years, which, in turn, facilitates the development of an effective warning system and appropriate allocation of resources for control and prevention.

The analysis demonstrated that the dengue case burden in the Philippines has remained persistently high over the past 8 years. Within the study period, the highest number of dengue cases was recorded in 2019, wherein more than 400,000 cases were reported in the country, translating into an incidence higher than in other ASEAN countries and China [6]. This surge in dengue cases was also observed globally. As reported by the World Health Organization [51], from the year 2000 to 2019, the number of reported dengue cases increased 10-fold from 500,000 to 5.2 million, with an unprecedented peak in 2019 [50]. The 2019 epidemic observed in the study warrants further investigation, but there are several factors known to be associated with dengue epidemics and increased risk of spread around the world which could explain the phenomenon. These include the expansion of the geographic distribution of vectors (primarily *Aedes aegypti*, and *Aedes albopictus*), especially in previously dengue-naive territories; and climate change factors such as rising environmental temperatures, high rainfall, and high humidity [5]. Among other factors are fragile health systems amid COVID-19 pandemic, political and financial instabilities in countries facing complex humanitarian crises, and high population movements [51]. Studies in the Philippines demonstrated the positive association between dengue incidence and climatic variables such as relative humidity [16], rainfall [17, 20, 52], and diurnal temperature range [52]. From 2020 to 2021, however, there was a substantial drop in the number of reported dengue cases. A phenomenon that was greatly influenced by the COVID-19 pandemic. As presented in the study of Seposo [53], the control measures implemented during the pandemic have reduced dengue transmission consequent to reduced population mobility, second, due to the fear of contracting COVID-19, people have developed the hesitancy of reporting by visiting health facilities. On a positive note, the pandemic has somehow impacted the health-seeking behavior of the population. The United Nations SDGs created a framework aiming to end the epidemics of neglected tropical diseases and malaria by 2030, but the country, along with the world is still far from meeting the milestones [6]. Hence, the country should pursue further the implementation of control measures and evaluate their effectiveness for improvement.

Geographically, dengue affects all the Philippine provinces with varying incidence. These results imply the widespread distribution of vectors across the country, requiring further entomological research for validation and hypothesis generation. In addition, further analyses such as hotspot analysis and space-time cluster analysis may be done to help fully understand the epidemiology of dengue. The varying incidence among the provinces may be explained by their inherent climatic variations, as dengue transmission can be affected by regional and local climatic fluctuations [54]. Simplistically, the Philippine climate consists of wet (December to April) and dry seasons (May to November) [34]. However, Corporal-Lodangco and Leslie [55] argue that the climate zones of the Philippines are more complex, with six climate zones identified and, in these zones, a province may belong in part or whole. Nevertheless, control and preventive measures must be implemented in the whole archipelago.

Time series analysis revealed strong seasonal variation of cases peaking between July and September annually. This similarly overlaps with neighboring countries such as Thailand [56], in which the cases peak in July to August, Vietnam [57], July to October, Cambodia [58], which extends through the wet season from May to October, and Lao PDR [59] with a peak occurring between June and September each year. However, in Singapore, outbreaks peak earlier between March and July [60]. This is due to climatic variations. In the Philippines, dengue incidence mostly occurs during the rainy season, and the amount of rainfall is strongly correlated to the increase of dengue cases [61]. Rainfall, humidity, and ambient temperature are considered macro factors that play a role in dengue transmission, as they have a direct impact on *Aedes aegypti* population density, which changes seasonally depending on these critical variables [62, 63]. Furthermore, mosquitoes were known to act as efficient transmitters within a relatively narrow temperature range [64], and variations in ambient temperature and humidity can lead to significant changes in vectorial capacity. Therefore, abrupt climatic changes, particularly the contrast between exceptionally hot years and milder ones, also affect transmission dynamics. This underscores the complex interplay between climatic variables and dengue transmission dynamics, emphasizing the need for the government to tailor surveillance and intervention strategies to the prevailing environmental conditions.

Regarding prediction, the NNAR model outperformed the other time series models evaluated in the study. This is attributed to its intrinsic ability to capture the complex nonlinear nature of the data series. This conforms with the study of Chakraborty et al. [21] where NNAR had superior performance over ARIMA, SVM, AR neural network, and LSTM models for short-term forecasts of dengue cases. Moreover, its hybrid model with ARIMA outperforms the other models and their hybrids for long-term forecasts. NNAR's superior performance was also found in forecasting other health outcomes, such as the incidence of tuberculosis in China [65] and India [66], hand-foot-and-mouth disease cases in China [67], the burden of esophageal cancer in China [68], mortality under 5 years old in Malaysia [69], and COVID-19 events in various countries, particularly the incidence in Pakistan [70], the second wave in Italy [71, 72] and the infection fatality rate in Brazil [73]. Although these findings are in agreement with the current study, different health outcomes may have differing data structures due to biological, temporal, and geographical variability, among other factors. It should be noted that the effectiveness of time-series methods depends on the underlying data characteristics and structure. In the Philippines, all four dengue serotypes circulate simultaneously [37], which confers a certain degree of stability to the data, unlike other settings where the introduction of a previously absent or long-unseen serotype introduces instability that presents difficulty in prediction, regardless of the forecasting model employed. Despite its merits, one disadvantage of the NNAR model is its declining accuracy with data characterized by intricate seasonal patterns [12]. Nonetheless, the above insights present the applicability of the NNAR model to various public health outcomes. Based on the model, a high number of cases the next 3 years is projected, which demands utmost vigilance. Furthermore, it is generally recommended that for continuous projections and better prediction, periodic reapplication of the time-series methodology using updated data is recommended at 6-month or yearly intervals [12].

The study implies further implementation of control and preventive measures. It also provides insights into how advanced forecasting models can be instrumental in creating benchmark predictions that assist authorities in setting pragmatic goals for the future. Perhaps an objective ensuring that dengue cases remain beneath the forecast values. To optimize prediction accuracy and timeliness, advanced forecasting approaches should be incorporated within established monitoring systems. Specifically, NNAR may be considered in developing a web-based early warning system that allows daily data updates from reporting hospital units and forecasts future outbreaks in the coming weeks or months in different regions of the Philippines.

The study differentiates from other studies, as it employed a univariate forecasting method to forecast dengue cases, which is less explored in other time series studies. It utilized more recent data and comparatively analyzed the performances of conventional and advanced statistical and machine learning time series methodologies. Furthermore, the study covered a wider geographic area than the previous forecasting studies in the country and was the first study thus far to apply TBATS and Prophet time series models in the Philippines.

The study's main limitation is probable imprecision, which stems from depending on data obtained from passive surveillance. Although the number of dengue cases in the Philippines is high, Edillo et al. [10] pointed out that less severe manifestations of dengue were underreported. Underreporting or nonreporting implies an underestimate of the true burden of the disease that could result in lower forecast estimates as a consequence. Nevertheless, the evidence of Edillo et al. [10] was derived from only one case study that requires to be reproduced around the country for validation. The surveillance system of the country is said to have greatly improved after the implementation of PIDSR-IS in 2007. Systematic reporting biases may occur at various organizational levels, especially since data quality relies heavily on each hospital's commitment to accurate reporting. Findings of the study may have limited generalizability beyond the Philippines due to variations in surveillance systems and case definitions across countries. Last, the study did not incorporate climatic variables or other established predictors of dengue outbreaks, which may have enhanced the models' predictive performance.

Despite these limitations, the forecast of the study still forewarns the government for future outbreaks. Further studies should investigate mapping techniques for risk assessment that incorporate environmental and climatic factors, utilizing approaches such as maximum entropy or ecological niche modeling as shown in earlier work [74]. Such approaches are justified in future research to enhance predictive accuracy and advance comprehension of spatial disease patterns and their environmental determinants. This study advanced the understanding of spatial and temporal epidemiology of dengue and the application of advanced time series methodologies for dengue forecasting in the Philippines.

## 5. Conclusions

To the best of the authors' knowledge, this is the first study to apply SARIMA, RF, LSTM, TBATS, and Prophet time series models in forecasting dengue cases in the Philippines at a national scale. The study provides a better understanding of the epidemiology of dengue in the Philippines in the aspect of geographic distribution, trend, seasonality, and forecast based on 8 years of data. NNAR stood out among the models considered in the study, which forecasted a high number of dengue cases in the coming years. This necessitates intensified control efforts to reduce such numbers and underscores the crucial role of incorporating forecasting in the existing surveillance system and policymaking. The approaches presented herein could be used by health agencies to forecast dengue outbreaks in short- or long-term periods. Thus, facilitates planning, design and improvement of control and preventive measures, along with proper resource allocation.

## Figures and Tables

**Table 1:** Error metrics for model evaluation.

Method	Training set				Testing set			
	MAE	MAPE	MASE	RMSE	MAE	MAPE	MASE	RMSE
SARIMA	3978.82	32.06	0.29	5821.71	16116.83	32.22	1.16	23719.20
NNAR	1256.81	8.13	0.09	2036.48	5506.36	13.23	0.40	8256.99
RF	2326.81	15.98	0.17	3801.72	10887.72	27.95	0.78	16322.85
LSTM	3329.36	30.50	0.24	4342.22	14905.57	52.86	1.07	18703.31
TBATS	2384.05	14.08	0.17	3306.83	16190.12	37.76	1.17	21862.34
Prophet	9396.86	89.39	0.68	13048.45	17129.33	40.23	1.23	23807.89

## Data Availability

The datasets presented in this article are not for public dissemination. An appropriate request must be made to the Epidemiology Bureau of the Department of Health, Philippines. Requests to access the datasets are directed to ebreports@doh.gov.ph.

## Supporting Information

Supporting Information 1 Table S1: Comparison of applications, assumptions, and limitations of forecasting models. Supporting Information Table S2: Annual rankings of provinces and major cities with high incidence (≥300 cases per 100,000 population). Supporting Information Table S3: Forecast values of dengue cases from 2025 to 2027 based on time series forecast models.

Supporting Information 2 Figure S1. ACF and PACF. Supporting Information Figure S2. Residuals of SARIMA model. Supporting Information Figure S3. Anomalies. Supporting Information Figure S4. Forecast of monthly dengue cases in the Philippines from 2025 to 2027 based on the time series models.
