# Supplementary material for: Spatial Distribution Analysis and Comparative Forecasting of Dengue Resurgence in the Philippines (2025–2027): A Nationwide Study
Source: Transbound Emerg Dis. 2025 Oct 15;2025:7480710. doi: 10.1155/tbed/7480710 (PMC12543447; doi:10.1155/tbed/7480710)
Supplement: Supporting Information 1 — Table S1: Comparison of applications, assumptions, and limitations of forecasting models. Supporting Information Table S2: Annual rankings of provinces and major cities with high incidence (≥300 cases per 100,000 population). Supporting Information Table S3: Forecast values of dengue cases from 2025 to 2027 based on time series forecast models. [file 7480710.f1.docx]

**Supplementary 2. Table**

**Supplement Table 1.** Comparison of applications, assumptions and limitations of forecasting models.

| **Model** | **Applications and assumptions** | **Limitations** |
| --- | --- | --- |
| **SARIMA** | - Suitable for univariate time series with clear seasonality and trend (Hyndman & Athanasopoulos, 2024; Pineda-Cortel et al., 2019)  - Assumes linear relationships (Punyapornwithaya et al., 2022)  - Requires stationary data or differencing (Hyndman & Athanasopoulos, 2024) | - Struggles with non-linear patterns  - Parameter tuning can be complex  - Poor performance on data with irregular seasonality |
| **NNAR** | - Captures non-linear patterns (Punyapornwithaya et al., 2023)  - Good for short- to medium-term forecasting  - Assumes lagged inputs represent time dependence (Hyndman & Athanasopoulos, 2024) | - Needs large data for training  - Lacks interpretability  - May overfit if not tuned carefully |
| **RF** | - Handles complex, non-linear relationships (Zhao et al., 2020).  - Can model interactions between variables  - Can be used with exogenous regressors (Olmoguez et al., 2019) | - Requires feature engineering for time series  - Poor extrapolation beyond training range  - Doesn’t model temporal autocorrelation directly |
| **LSTM** | - Suitable for long-term dependencies in time series (Ligue & Ligue, 2022)  - Effective for sequential and non-linear data  - Works on multivariate and univariate data | - Computationally intensive  - Requires large datasets and careful tuning  - Less interpretable than traditional models |
| **TBATS** | - Handles multiple seasonalities and complex seasonal patterns (Hyndman & Athanasopoulos, 2024)  - Works with high-frequency time series  - Built-in Box-Cox transformation and ARMA errors | - Can be computationally expensive  - Less accurate for very noisy or irregular data  - May not generalize well to all domains |
| **Prophet** | - Suitable for time series that have strong seasonality and several seasons of historical data (Taylor & Letham, 2018).  - Handles missing data, holidays, and seasonality (Hyndman & Athanasopoulos, 2024)  - Assumes additive or multiplicative components | - Assumes trend and seasonality are smooth  - May underperform on high-frequency or highly irregular data  - Less flexible for highly non-linear dynamics |

**References:**

Hyndman, R. J., & Athanasopoulos, G. (2024). *Forecasting: principles and practice* (3rd ed.). OTexts. <https://otexts.com/fpp3/>

Ligue, K. D. B., & Ligue, K. J. B. (2022). Deep Learning Approach to Forecasting Dengue Cases in Davao City Using Long Short-term Memory (LSTM) [Article]. *Philippine Journal of Science*, *151*(3), 797-812. <https://www.scopus.com/inward/record.uri?eid=2-s2.0-85131794442&partnerID=40&md5=a49862688e71965468b3f8f484a1bb59>

Olmoguez, I. L. G., Catindig, M. A. C., Amongos, M. F. L., & Lazan, F. G. (2019). Developing a dengue forecasting model: A case study in Iligan City [Article]. *International Journal of Advanced Computer Science and Applications*, *10*(9), 281-286. <https://doi.org/10.14569/ijacsa.2019.0100936>

Pineda-Cortel, M. R. B., Clemente, B. M., & Nga, P. T. T. (2019). Modeling and predicting dengue fever cases in key regions of the Philippines using remote sensing data [Article]. *Asian Pacific Journal of Tropical Medicine*, *12*(2), 60-66. <https://doi.org/10.4103/1995-7645.250838>

Punyapornwithaya, V., Mishra, P., Sansamur, C., Pfeiffer, D., Arjkumpa, O., Prakotcheo, R., Damrongwatanapokin, T., & Jampachaisri, K. (2022). Time-Series Analysis for the Number of Foot and Mouth Disease Outbreak Episodes in Cattle Farms in Thailand Using Data from 2010–2020. *Viruses*, *14*(7), 1367. <https://doi.org/https://doi.org/10.3390/v14071367>

Punyapornwithaya, V., Thanapongtharm, W., Jainonthee, C., Chinsorn, P., Sagarasaeranee, O., Salvador, R., & Arjkumpa, O. (2023). Time series analysis and forecasting of the number of canine rabies confirmed cases in Thailand based on national-level surveillance data [Article]. *Frontiers in veterinary science*, *10*, Article 1294049. <https://doi.org/10.3389/fvets.2023.1294049>

Taylor, S. J., & Letham, B. (2018). Forecasting at scale. The American Statistician, 72(1), 37–45. <https://doi.org/10.1080/00031305.2017.1380080>

Zhao, N., Charland, K., Carabali, M., Nsoesie, E. O., Maheu-Giroux, M., Rees, E., Yuan, M., Balaguera, C. G., Ramirez, G. J., & Zinszer, K. (2020). Machine learning and dengue forecasting: Comparing random forests and artificial neural networks for predicting dengue burden at national and sub-national scales in Colombia [Article]. *PLOS Neglected Tropical Diseases*, *14*(9), 1-16, Article e0008056. <https://doi.org/10.1371/journal.pntd.0008056>

**Supplement Table 2.** Annual rankings of provinces and major cities with high incidence (≥ 300 cases per 100,000 population)

| **Rank** | **2017** | | **2018** | | **2019** | |
| --- | --- | --- | --- | --- | --- | --- |
|  | **Province/city** | **Incidence** | **Province/city** | **Incidence** | **Province/city** | **Incidence** |
| 1 | Kalinga | 698.49 | Batanes | 6576.65 | Guimaras | 1349.98 |
| 2 | Zambales | 470.19 | Apayao | 1566.35 | Iloilo | 1178.17 |
| 3 | City Of Isabela | 403.84 | Quirino | 1156.09 | Tacloban City | 1136.45 |
| 4 | Naga City | 385.15 | Camiguin | 934.64 | Apayao | 1117.79 |
| 5 | Bacolod City | 344.07 | Nueva Vizcaya | 769.12 | Zamboanga Sibugay | 994.42 |
| 6 | Quezon City | 335.66 | Kalinga | 727.33 | Aklan | 981.06 |
| 7 | General Santos City (Dadiangas) | 329.20 | Misamis Occidental | 630.57 | Quirino | 934.95 |
| 8 | South Cotabato | 325.38 | Surigao Del Sur | 546.46 | Samar (Western Samar) | 921.87 |
| 9 | Bataan | 306.01 | Bukidnon | 515.98 | Capiz | 868.73 |
| 10 | City Of San Juan | 302.81 | Puerto Princesa City | 508.40 | Camiguin | 865.41 |
| 11 |  |  | Tarlac | 482.92 | Zamboanga City | 822.85 |
| 12 |  |  | Lapu-Lapu City (Opon) | 474.26 | City Of Isabela | 818.13 |
| 13 |  |  | City Of Santiago | 470.30 | Eastern Samar | 807.68 |
| 14 |  |  | Abra | 444.67 | Iloilo City | 751.28 |
| 15 |  |  | Isabela | 437.07 | Nueva Vizcaya | 712.60 |
| 16 |  |  | Cagayan De Oro City | 431.12 | Kalinga | 710.49 |
| 17 |  |  | Aurora | 428.22 | South Cotabato | 706.97 |
| 18 |  |  | Ifugao | 425.29 | Laguna | 706.26 |
| 19 |  |  | City Of Isabela | 405.61 | Aurora | 658.68 |
| 20 |  |  | Bohol | 400.36 | Bukidnon | 653.65 |
| 21 |  |  | Surigao Del Norte | 388.89 | Ilocos Sur | 621.15 |
| 22 |  |  | Cebu City | 368.18 | Dinagat Islands | 610.28 |
| 23 |  |  | Eastern Samar | 361.63 | Mountain Province | 608.22 |
| 24 |  |  | Mandaue City | 355.53 | Cagayan De Oro City | 595.32 |
| 25 |  |  | City Of Paranaque | 355.24 | Cavite | 575.60 |
| 26 |  |  | Misamis Oriental | 342.39 | Lanao Del Norte | 573.98 |
| 27 |  |  | Aklan | 339.91 | Iligan City | 572.46 |
| 28 |  |  | South Cotabato | 339.42 | Ifugao | 567.79 |
| 29 |  |  | Agusan Del Sur | 336.37 | Misamis Oriental | 562.52 |
| 30 |  |  | Nueva Ecija | 326.48 | Abra | 546.79 |
| 31 |  |  | Lanao Del Norte | 323.39 | Bohol | 539.17 |
| 32 |  |  | Samar (Western Samar) | 320.68 | Quezon | 535.43 |
| 33 |  |  | Ilocos Norte | 316.05 | Zamboanga Del Norte | 531.89 |
| 34 |  |  | La Union | 312.72 | Tarlac | 527.03 |
| 35 |  |  | Negros Occidental | 311.47 | Surigao Del Norte | 526.09 |
| 36 |  |  | Bataan | 311.19 | Ormoc City | 514.95 |
| 37 |  |  | Quezon City | 308.63 | Antique | 513.26 |
| 38 |  |  | Siquijor | 304.44 | Pateros | 505.65 |
| 39 |  |  | Northern Samar | 302.09 | Misamis Occidental | 499.07 |
| 40 |  |  | Butuan City | 301.38 | City Of Paranaque | 491.41 |
| 41 |  |  |  |  | Siquijor | 489.61 |
| 42 |  |  |  |  | Negros Oriental | 484.71 |
| 43 |  |  |  |  | Ilocos Norte | 477.11 |
| 44 |  |  |  |  | Biliran | 476.54 |
| 45 |  |  |  |  | Batangas | 471.81 |
| 46 |  |  |  |  | Bacolod City | 470.65 |
| 47 |  |  |  |  | Butuan City | 462.44 |
| 48 |  |  |  |  | Lapu-Lapu City (Opon) | 458.75 |
| 49 |  |  |  |  | Negros Occidental | 457.51 |
| 50 |  |  |  |  | Marinduque | 455.54 |
| 51 |  |  |  |  | Agusan Del Norte | 452.00 |
| 52 |  |  |  |  | Zamboanga Del Sur | 450.06 |
| 53 |  |  |  |  | Surigao Del Sur | 446.93 |
| 54 |  |  |  |  | Cagayan | 445.77 |
| 55 |  |  |  |  | Naga City | 445.50 |
| 56 |  |  |  |  | Cotabato (North Cotabato) | 443.93 |
| 57 |  |  |  |  | City Of Makati | 428.85 |
| 58 |  |  |  |  | Puerto Princesa City | 426.03 |
| 59 |  |  |  |  | Agusan Del Sur | 417.58 |
| 60 |  |  |  |  | Leyte | 411.01 |
| 61 |  |  |  |  | City Of Malabon | 405.10 |
| 62 |  |  |  |  | Sarangani | 394.33 |
| 63 |  |  |  |  | Catanduanes | 392.67 |
| 64 |  |  |  |  | Cebu City | 388.33 |
| 65 |  |  |  |  | City Of Pasig | 385.82 |
| 66 |  |  |  |  | Quezon City | 379.87 |
| 67 |  |  |  |  | Nueva Ecija | 368.97 |
| 68 |  |  |  |  | Bataan | 367.22 |
| 69 |  |  |  |  | City Of San Juan | 364.57 |
| 70 |  |  |  |  | Benguet | 363.30 |
| 71 |  |  |  |  | Palawan | 357.76 |
| 72 |  |  |  |  | Cebu | 357.38 |
| 73 |  |  |  |  | Oriental Mindoro | 354.94 |
| 74 |  |  |  |  | Isabela | 348.39 |
| 75 |  |  |  |  | Sultan Kudarat | 347.67 |
| 76 |  |  |  |  | Northern Samar | 341.87 |
| 77 |  |  |  |  | Rizal | 330.59 |
| 78 |  |  |  |  | Mandaue City | 322.67 |
| 79 |  |  |  |  | Pangasinan | 321.65 |
| 80 |  |  |  |  | Dagupan City | 319.58 |
| 81 |  |  |  |  | Occidental Mindoro | 315.56 |
| 82 |  |  |  |  | City Of Muntinlupa | 312.14 |
| 83 |  |  |  |  | Taguig City | 311.34 |
| 84 |  |  |  |  | Cotabato City | 309.86 |
| 85 |  |  |  |  | General Santos City (Dadiangas) | 302.48 |
|  | | | | | | |
| **Rank** | **2020** | | **2021** | | **2022** | |
|  | **Province/city** | **Incidence** | **Province/city** | **Incidence** | **Province/city** | **Incidence** |
| 1 | Puerto Princesa City | 368.72 | Baguio City | 368.46 | Apayao | 1888.77 |
| 2 | Siquijor | 360.39 | Kalinga | 309.13 | Kalinga | 1595.34 |
| 3 |  |  |  |  | Baguio City | 1017.37 |
| 4 |  |  |  |  | Nueva Vizcaya | 980.57 |
| 5 |  |  |  |  | Occidental Mindoro | 758.59 |
| 6 |  |  |  |  | Ifugao | 757.06 |
| 7 |  |  |  |  | Aurora | 700.21 |
| 8 |  |  |  |  | Benguet | 573.05 |
| 9 |  |  |  |  | Mountain Province | 551.32 |
| 10 |  |  |  |  | Cagayan | 540.37 |
| 11 |  |  |  |  | Nueva Ecija | 534.27 |
| 12 |  |  |  |  | City Of Malabon | 529.20 |
| 13 |  |  |  |  | Batanes | 518.05 |
| 14 |  |  |  |  | City Of Valenzuela | 512.87 |
| 15 |  |  |  |  | Dinagat Islands | 475.66 |
| 16 |  |  |  |  | Bulacan | 473.48 |
| 17 |  |  |  |  | Zamboanga City | 465.13 |
| 18 |  |  |  |  | Quirino | 451.02 |
| 19 |  |  |  |  | Cotabato City | 434.71 |
| 20 |  |  |  |  | Antique | 425.78 |
| 21 |  |  |  |  | City Of Isabela | 415.66 |
| 22 |  |  |  |  | Isabela | 400.88 |
| 23 |  |  |  |  | Pateros | 398.48 |
| 24 |  |  |  |  | City Of Santiago | 378.34 |
| 25 |  |  |  |  | Camiguin | 352.03 |
| 26 |  |  |  |  | Southern Leyte | 350.79 |
| 27 |  |  |  |  | Biliran | 346.25 |
| 28 |  |  |  |  | Lapu-Lapu City (Opon) | 342.00 |
| 29 |  |  |  |  | Cebu City | 340.12 |
| 30 |  |  |  |  | Surigao Del Norte | 332.54 |
| 31 |  |  |  |  | Taguig City | 323.41 |
| 32 |  |  |  |  | Puerto Princesa City | 312.19 |
| 33 |  |  |  |  | Ilocos Norte | 311.49 |
| 34 |  |  |  |  | Tarlac | 310.98 |
| 35 |  |  |  |  | Pampanga | 310.63 |
| 36 |  |  |  |  | Zamboanga Sibugay | 306.50 |
| 37 |  |  |  |  | Naga City | 300.84 |
|  | | | | | | |
| **Rank** | **2023** | | **2024** | |  | |
|  | **Province/city** | **Incidence** | **Province/city** | **Incidence** |  |  |
| 1 | Batanes | 2411.50 | Baguio City | 2327.48 |  |  |
| 2 | Ifugao | 1308.45 | Mountain Province | 2258.11 |  |  |
| 3 | Puerto Princesa City | 1094.74 | Apayao | 1560.39 |  |  |
| 4 | Nueva Vizcaya | 835.61 | Benguet | 1264.62 |  |  |
| 5 | Mountain Province | 646.11 | Quezon City | 1250.17 |  |  |
| 6 | Apayao | 635.33 | Siquijor | 1209.27 |  |  |
| 7 | Palawan | 582.74 | Kalinga | 1164.84 |  |  |
| 8 | General Santos City (Dadiangas) | 580.49 | Guimaras | 1147.35 |  |  |
| 9 | Sarangani | 491.04 | Ifugao | 1075.53 |  |  |
| 10 | Davao De Oro | 458.02 | City Of Isabela | 995.63 |  |  |
| 11 | Davao Oriental | 456.13 | Nueva Vizcaya | 947.65 |  |  |
| 12 | Zamboanga Del Norte | 440.33 | Surigao Del Norte | 892.75 |  |  |
| 13 | Kalinga | 434.13 | Davao Del Norte | 824.71 |  |  |
| 14 | Davao Del Norte | 426.61 | Quirino | 780.89 |  |  |
| 15 | Cotabato City | 419.97 | Aurora | 684.14 |  |  |
| 16 | Iligan City | 400.40 | Surigao Del Sur | 673.91 |  |  |
| 17 | Siquijor | 398.88 | Bohol | 649.93 |  |  |
| 18 | Misamis Occidental | 396.81 | Bukidnon | 613.47 |  |  |
| 19 | City Of Isabela | 392.62 | Davao De Oro | 612.85 |  |  |
| 20 | Pateros | 392.32 | Oriental Mindoro | 600.50 |  |  |
| 21 | Baguio City | 389.03 | Tarlac | 587.33 |  |  |
| 22 | Romblon | 364.52 | Dinagat Islands | 567.73 |  |  |
| 23 | Davao City | 363.46 | Puerto Princesa City | 553.06 |  |  |
| 24 | Tarlac | 360.14 | Misamis Occidental | 542.47 |  |  |
| 25 | City Of Malabon | 351.61 | Caloocan City | 525.99 |  |  |
| 26 | Ilocos Norte | 344.77 | Batanes | 523.99 |  |  |
| 27 | Bukidnon | 341.23 | Iloilo City | 523.96 |  |  |
| 28 | Lanao Del Norte | 320.15 | Butuan City | 516.95 |  |  |
| 29 |  |  | Aklan | 508.62 |  |  |
| 30 |  |  | Cotabato (North Cotabato) | 500.24 |  |  |
| 31 |  |  | Sarangani | 496.12 |  |  |
| 32 |  |  | Palawan | 490.47 |  |  |
| 33 |  |  | Cebu City | 474.07 |  |  |
| 34 |  |  | Romblon | 468.16 |  |  |
| 35 |  |  | Iloilo | 465.89 |  |  |
| 36 |  |  | Lanao Del Sur | 462.42 |  |  |
| 37 |  |  | Davao Occidental | 460.93 |  |  |
| 38 |  |  | City Of Santiago | 456.96 |  |  |
| 39 |  |  | Zamboanga Del Norte | 453.87 |  |  |
| 40 |  |  | Dagupan City | 451.95 |  |  |
| 41 |  |  | Lapu-Lapu City (Opon) | 441.27 |  |  |
| 42 |  |  | Iligan City | 439.58 |  |  |
| 43 |  |  | Occidental Mindoro | 439.45 |  |  |
| 44 |  |  | City Of Pasig | 435.07 |  |  |
| 45 |  |  | General Santos City (Dadiangas) | 433.05 |  |  |
| 46 |  |  | Sultan Kudarat | 424.72 |  |  |
| 47 |  |  | Bacolod City | 419.96 |  |  |
| 48 |  |  | Capiz | 418.92 |  |  |
| 49 |  |  | Negros Oriental | 411.62 |  |  |
| 50 |  |  | Taguig City | 394.56 |  |  |
| 51 |  |  | Ormoc City | 386.11 |  |  |
| 52 |  |  | Zamboanga Sibugay | 379.44 |  |  |
| 53 |  |  | Mandaue City | 372.64 |  |  |
| 54 |  |  | Davao City | 370.13 |  |  |
| 55 |  |  | Rizal | 370.05 |  |  |
| 56 |  |  | South Cotabato | 358.76 |  |  |
| 57 |  |  | Marinduque | 356.71 |  |  |
| 58 |  |  | Antique | 349.74 |  |  |
| 59 |  |  | Bulacan | 339.50 |  |  |
| 60 |  |  | Cagayan | 337.07 |  |  |
| 61 |  |  | Camiguin | 335.21 |  |  |
| 62 |  |  | Cavite | 333.69 |  |  |
| 63 |  |  | La Union | 329.43 |  |  |
| 64 |  |  | City Of Manila | 326.66 |  |  |
| 65 |  |  | Zamboanga Del Sur | 317.77 |  |  |
| 66 |  |  | City Of Marikina | 316.08 |  |  |
| 67 |  |  | Agusan Del Norte | 314.33 |  |  |
| 68 |  |  | Maguindanao Del Norte | 311.97 |  |  |

Incidence = (no. of cases / midyear population) x 100,000 population

**Supplement Table 3.** Forecast values of dengue cases from 2025 to 2027 based on time series forecast models.

| **Year** | **Month** | **SARIMA** | **NNAR** | **RF** | **LSTM** | **TBATS** | **Prophet** | **Mean** |
| --- | --- | --- | --- | --- | --- | --- | --- | --- |
| 2025 | Jan | 30375 | 27476 | 34467 | 26874 | 37154 | 17377 | 28953.64 |
| 2025 | Feb | 26242 | 23244 | 38723 | 22872 | 27188 | 14563 | 25471.88 |
| 2025 | Mar | 23813 | 20485 | 43580 | 19688 | 21292 | 14329 | 23864.25 |
| 2025 | Apr | 21404 | 17940 | 47688 | 17332 | 15946 | 12826 | 22189.22 |
| 2025 | May | 21044 | 17058 | 53515 | 15672 | 19444 | 15568 | 23716.86 |
| 2025 | Jun | 24371 | 17981 | 61078 | 15642 | 34908 | 22926 | 29484.23 |
| 2025 | Jul | 36408 | 33478 | 66265 | 18927 | 60107 | 37343 | 42087.96 |
| 2025 | Aug | 47434 | 81297 | 66099 | 27301 | 68964 | 44386 | 55913.43 |
| 2025 | Sep | 35995 | 84169 | 66019 | 40303 | 50160 | 33855 | 51750.22 |
| 2025 | Oct | 31471 | 53448 | 65892 | 47237 | 46052 | 29153 | 45542.10 |
| 2025 | Nov | 30295 | 39546 | 65847 | 38219 | 41527 | 26791 | 40370.83 |
| 2025 | Dec | 26257 | 28342 | 65791 | 31371 | 31736 | 21168 | 34110.79 |
| 2026 | Jan | 25012 | 22997 | 65505 | 26183 | 36258 | 21109 | 32843.79 |
| 2026 | Feb | 23259 | 20913 | 65705 | 22113 | 27244 | 16851 | 29347.53 |
| 2026 | Mar | 22217 | 20031 | 65695 | 19271 | 21248 | 14826 | 27214.48 |
| 2026 | Apr | 21197 | 18923 | 65694 | 17000 | 15978 | 12576 | 25227.89 |
| 2026 | May | 21021 | 18236 | 65681 | 15559 | 19405 | 15212 | 25852.47 |
| 2026 | Jun | 22352 | 17703 | 65681 | 16006 | 34977 | 23972 | 30115.20 |
| 2026 | Jul | 27229 | 28067 | 65681 | 20083 | 59989 | 40798 | 40307.84 |
| 2026 | Aug | 31699 | 66747 | 65681 | 29405 | 69098 | 45385 | 51335.78 |
| 2026 | Sep | 27039 | 95466 | 65681 | 42457 | 50063 | 35072 | 52629.80 |
| 2026 | Oct | 25194 | 63970 | 65681 | 46053 | 46140 | 29777 | 46135.81 |
| 2026 | Nov | 24711 | 45532 | 65681 | 36538 | 41449 | 26133 | 40007.34 |
| 2026 | Dec | 23066 | 34359 | 65681 | 30304 | 31795 | 21184 | 34398.29 |
| 2027 | Jan | 22556 | 24476 | 65681 | 25280 | 36191 | 24882 | 33177.71 |
| 2027 | Feb | 21842 | 21832 | 65681 | 21499 | 27294 | 19102 | 29541.71 |
| 2027 | Mar | 21417 | 19772 | 65681 | 18793 | 21210 | 15275 | 27024.41 |
| 2027 | Apr | 21001 | 19115 | 65681 | 16638 | 16007 | 12309 | 25125.37 |
| 2027 | May | 20929 | 18410 | 65681 | 15454 | 19371 | 14895 | 25789.96 |
| 2027 | Jun | 21469 | 17933 | 65681 | 16451 | 35038 | 25085 | 30276.19 |
| 2027 | Jul | 23451 | 22574 | 65681 | 21444 | 59885 | 44254 | 39547.99 |
| 2027 | Aug | 25268 | 46448 | 65681 | 31800 | 69217 | 46340 | 47458.98 |
| 2027 | Sep | 23374 | 90804 | 65681 | 44557 | 49978 | 36261 | 51775.81 |
| 2027 | Oct | 22623 | 72225 | 65681 | 44408 | 46218 | 30372 | 46921.20 |
| 2027 | Nov | 22427 | 45322 | 65681 | 35074 | 41380 | 25479 | 39227.17 |
| 2027 | Dec | 21758 | 37718 | 65681 | 29275 | 31848 | 21276 | 34592.71 |
