# Supplementary material for: Spatial Distribution Analysis and Comparative Forecasting of Dengue Resurgence in the Philippines (2025–2027): A Nationwide Study
Source: Transbound Emerg Dis. 2025 Oct 15;2025:7480710. doi: 10.1155/tbed/7480710 (PMC12543447; doi:10.1155/tbed/7480710)
Supplement: Supporting Information 2 — Figure S1. ACF and PACF. Supporting Information Figure S2. Residuals of SARIMA model. Supporting Information Figure S3. Anomalies. Supporting Information Figure S4. Forecast of monthly dengue cases in the Philippines from 2025 to 2027 based on the time series models. [file 7480710.f2.docx]

**Supplementary 1. Figures**


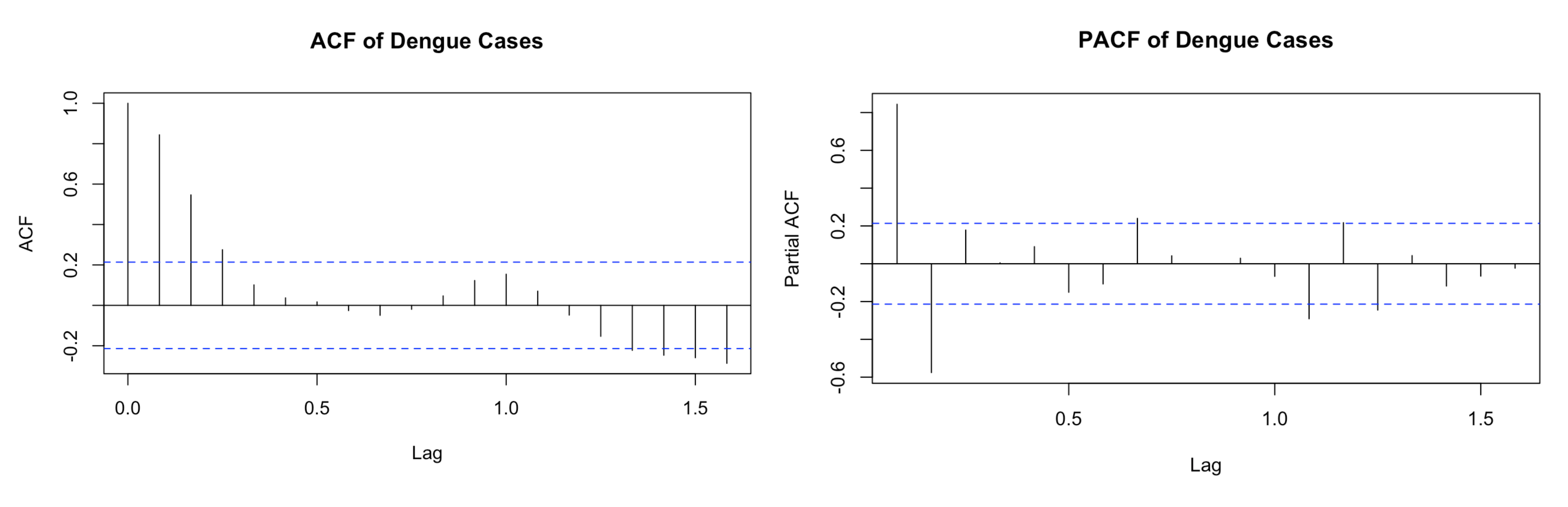


**Supplement Figure 1.** ACF and PACF


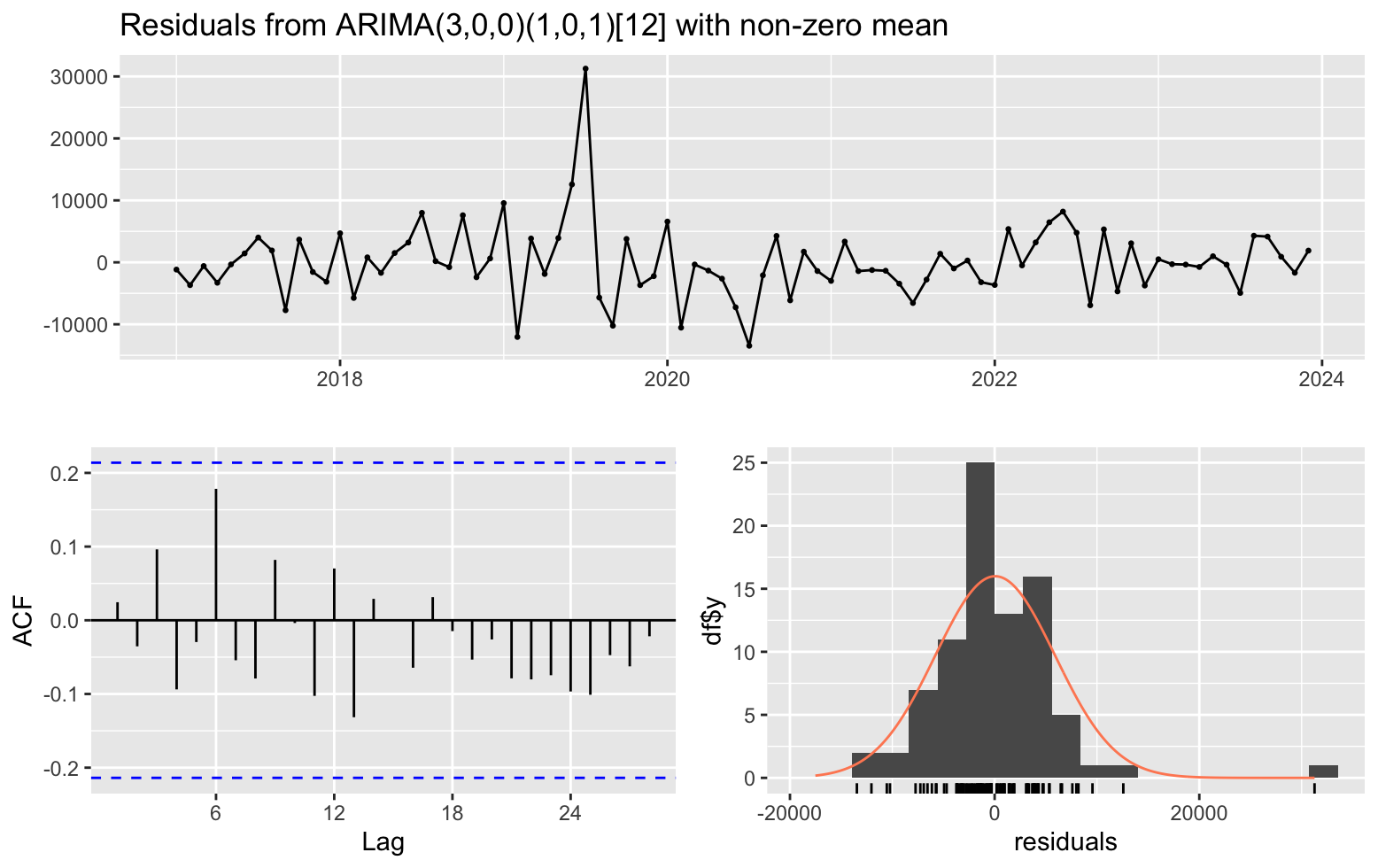


**Supplement Figure 2.** Residuals of SARIMA model


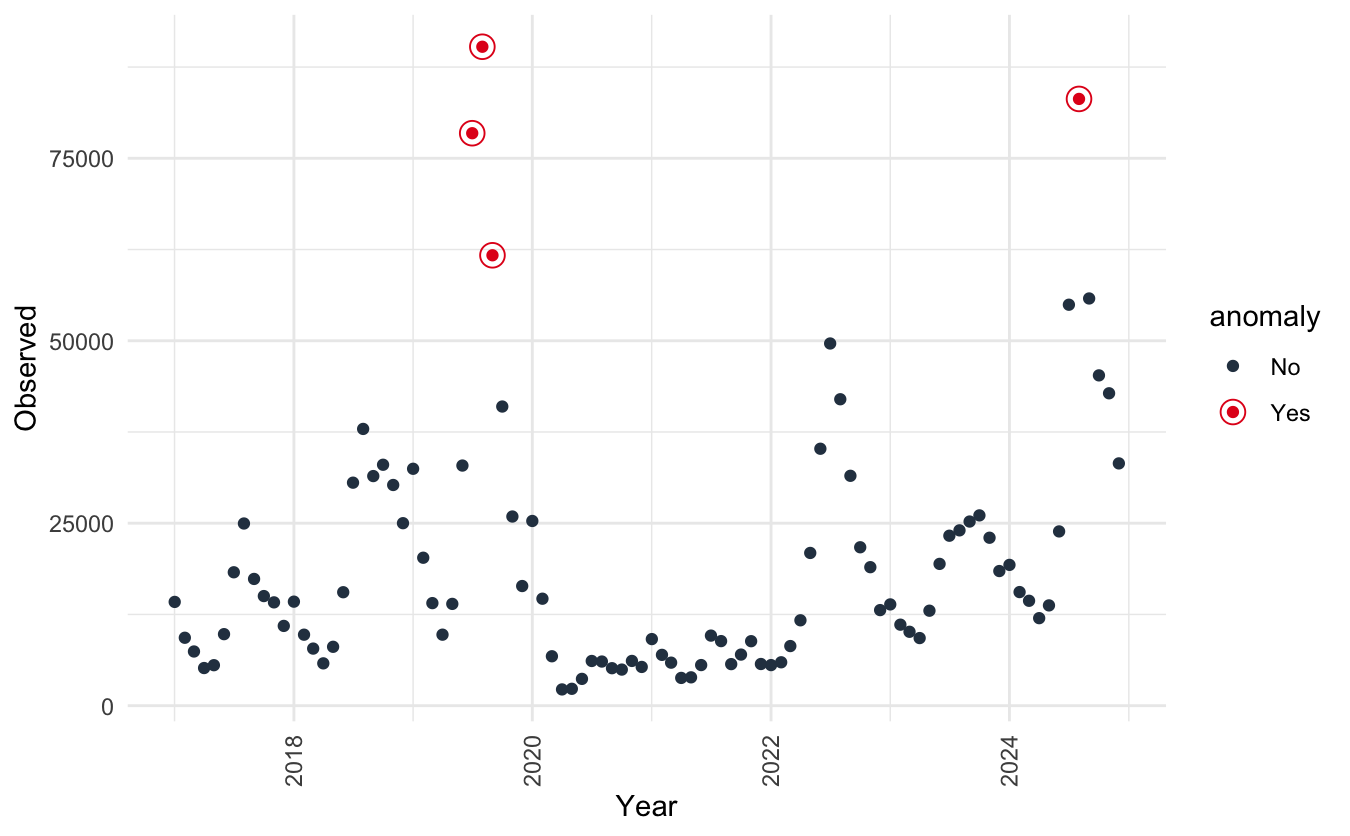


**Supplement Figure 3.** Anomalies


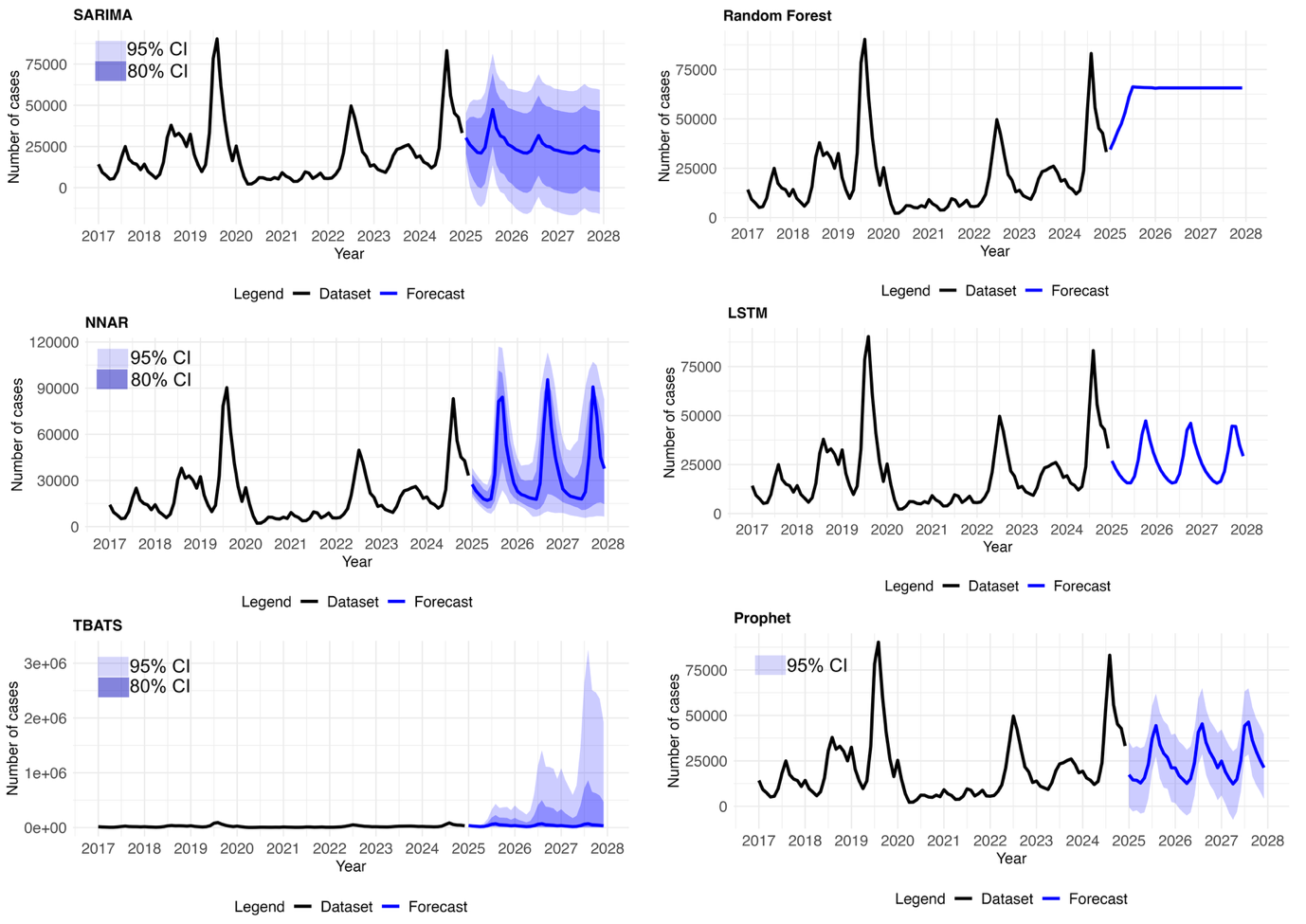


**Supplement Figure 4.** Forecast of monthly dengue cases in the Philippines from 2025 to 2027 based on the time series models.
